# Positive association between dietary acid load and future insulin resistance risk: findings from the Korean Genome and Epidemiology Study

**DOI:** 10.1186/s12937-020-00653-6

**Published:** 2020-12-08

**Authors:** Kyung Won Lee, Dayeon Shin

**Affiliations:** 1 Department of Home Economics Education, Korea National University of Education, Cheongju, 28173 South Korea; 2 Department of Food and Nutrition, Inha University, 100 Inha-ro, Michuhol-gu, Incheon, 22212 South Korea

**Keywords:** Dietary acid load, Acid-base balance, Diet-induced metabolic acidosis, Insulin resistance, Prospective study, Koreans

## Abstract

**Background:**

Acid-base homeostasis is increasingly being recognized to play an important role in normal metabolic function. However, prospective studies on the relationship between diet-induced acid-base imbalance and insulin resistance among Asian populations have been limited. Thus, we investigated whether diet-induced metabolic acidosis was prospectively associated with insulin resistance risk in middle-aged and older Korean adults.

**Methods:**

In total, 5406 participants from the Korean Genome and Epidemiology Study without type 2 diabetes, insulin resistance, cancer, or renal diseases at baseline examination were included in this study. To estimate diet-induced metabolic acidosis, we used potential renal acid load (PRAL) and net endogenous acid production (NEAP) scores calculated from the usual dietary intake assessed by a validated 103-item food frequency questionnaire at baseline. Multivariable Cox proportional hazard models were applied to estimate hazard ratios (HRs) and 95% confidence intervals (CIs) of insulin resistance incidence.

**Results:**

During a mean follow-up period of 7.4 years, we documented 3449 insulin resistance cases. In the fully adjusted model, the future insulin resistance risk was significantly higher among participants in the highest quartiles of PRAL (HR: 1.30, 95% CI: 1.13–1.48, *P*_trend_ = 0.0002) and NEAP (HR: 1.30, 95% CI: 1.14–1.49, *P*_trend_ = 0.0008) than among those in the lowest quartiles. Associations were slightly strengthened among men, adults < 50 years old, obese participants, or those with low physical activity levels.

**Conclusions:**

Our findings suggested that diet-dependent acid load was positively associated with the future development of insulin resistance, suggesting effect modification by sex, age, the presence of obesity, and physical activity levels.

**Supplementary Information:**

The online version contains supplementary material available at 10.1186/s12937-020-00653-6.

## Introduction

Acid-base balance in the body is crucial for sustaining metabolic health. In a healthy person, acid-base homeostasis is maintained by the lungs, kidneys, and various chemical buffering systems [1]. Among the previously investigated determinants of acid-base balance, diet was a major contributing factor to body acidity and alkalinity [2, 3]. Particularly, it is known that Western-style diets are more prone to acid-base abnormalities. Typical Western-style diets are considered to have high acid-forming potential due to high acidogenic food consumption (meat and processed wheat-based products) and low alkalizing food consumption (vegetables and fruits) [4, 5]. Habitually skewed consumption of highly acidic or alkaline foods may result in chronic acid-base imbalance in the body, which in turn causes adverse metabolic conditions [6]; however, the underlying mechanisms are poorly understood.

To estimate the potential acid load produced by a single food or overall diet, two validated measurements, potential renal acid load (PRAL) [7] and net endogenous acid production (NEAP) [8], are commonly used. The PRAL score is based on intake of protein, phosphorus, potassium, magnesium, and calcium [7], while the NEAP score is based on protein and potassium intake, which are the main determinants of endogenous acid production [8]. Higher PRAL and NEAP scores indicate diets with high acid-forming potential.

Previous studies have suggested that acid-base imbalance, estimated by dietary acid load scores, is associated with unfavorable metabolic symptoms such as abdominal obesity [9, 10], hypertension [11], and abnormal lipid profiles [12]. In Western countries following high dietary protein intake, diet-induced metabolic acidosis is associated with increased risks of insulin resistance and type 2 diabetes [13–15]. However, to date, little is known about these associations in Korean populations. Given that substantial differences in intake quantities and sources of dietary protein exist between Asian and Western populations [16], studies investigating the effects of diet-induced acid-base imbalance on insulin resistance among Koreans are warranted.

Therefore, using two dietary acid load scores to estimate the acid-forming potential of Korean diets, we tested whether diet-induced metabolic acidosis was associated with the future risk of insulin resistance and whether this relationship differed with effect modifiers among the middle-aged and elderly Korean population.

## Methods

### Data source and study population

The Korean Genome and Epidemiology Study (KoGES) is an ongoing, population-based, prospective study run by the Korea National Institute of Health. In 2001, as part of the KoGES, the Ansan-Ansung Cohort Study began recruiting 10,030 Korean adults between 40 and 69 years old living in urban (Ansan) and rural (Ansung) communities in Korea, and participants were followed biennially. During the baseline and follow-up examinations, data on demographic factors, lifestyle, disease and medication histories, and reproductive health were obtained from structured questionnaires by trained interviewers to examine the effects of dietary, lifestyle, and environmental factors on chronic disease in Koreans. Blood and urine samples were collected from participants during visits at examination centers and analyzed at a certified laboratory with standard protocols.

**Fig. 1:**
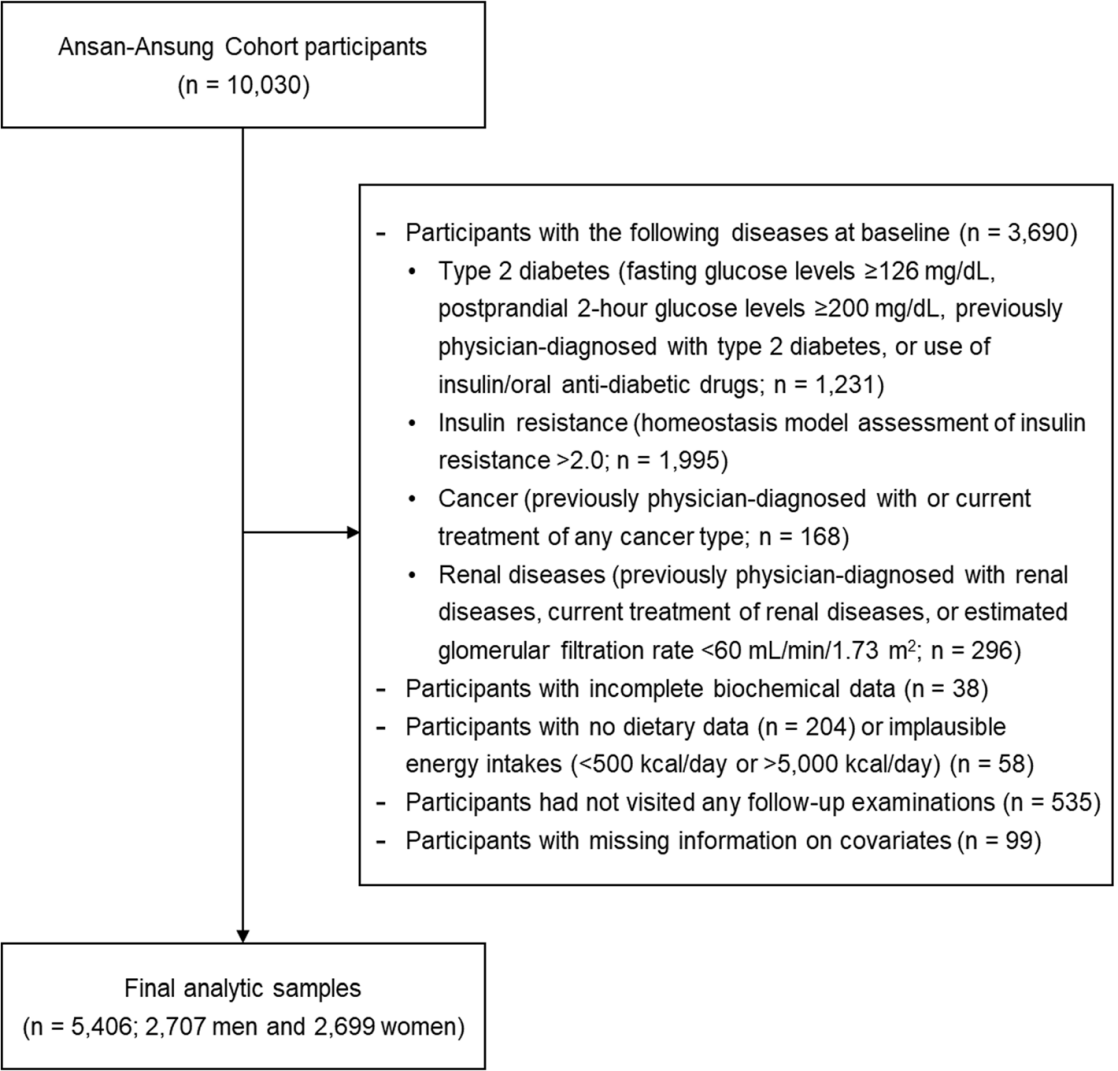
Flowchart of the study population

For the current study, we excluded participants with type 2 diabetes (*n* = 1231), insulin resistance (*n* = 1995), cancer (*n* = 168), or renal diseases (*n* = 296) at baseline examination (Fig. 1). Of the remaining 6340 participants, those with incomplete data were further excluded: missing biochemical (*n* = 38) or dietary (*n* = 204) data values, implausible energy intakes (< 500 kcal/day or > 5000 kcal/day) [17–19] (*n* = 58), no follow-up visits after baseline examination (*n* = 535), and missing covariate information (*n* = 99). The final analytic sample was 5406 participants (2707 men and 2699 women), and data were obtained from follow-up examinations until 2016. The KoGES study was reviewed and approved by the Institutional Review Board of the Korea Centers for Disease Control and Prevention, and all participants provided informed consent. All study methods and protocols were conducted in accordance with the relevant institutional guidelines and regulations.

### Dietary assessment and dietary acid load score

Dietary intake was measured at baseline examination using a validated 103-item semi-quantitative food frequency questionnaire (FFQ). The FFQ was developed to capture the usual food and nutrient intake of KoGES participants during the preceding year and validated against four 3-day dietary records [20]. The FFQ included nine frequency categories (ranging from “never/rarely” to “more than 3 times a day”) and three portion size categories (“small: one half of the standard portion size,” “medium: one standard portion size,” and “large: two standard portion sizes”) for each food and beverage item. To estimate the daily food and nutrient intake, the consumption frequency of each food and beverage was multiplied by its energy and nutrient contents in one portion size, and contributions from all foods and beverages were summed. Information on nutritive values of all foods and beverages listed in the FFQ was obtained from the Korean Food Composition Table [21].

Dietary acid load for each participant was estimated from two scores: PRAL, which measures the intestinal absorption rates of protein, potassium, calcium, and magnesium and dissociation of phosphate at pH 7.4 [7], and NEAP, which measures the acidifying potential of protein and alkalizing potential of potassium from consumed foods [8]. PRAL and NEAP scores were derived from the following formulas:

PRAL (mEq/day) = [protein (g/d) × 0.49 + phosphorus (mg/d) × 0.037] - [potassium (mg/d) × 0.0211 - magnesium (mg/d) × 0.0263 - calcium (mg/d) × 0.013] [7].

NEAP (mEq/day) = [protein (g/d) × 54.5 / potassium (mEq/day)] - 10.2 [8].

To compute the dietary acid load scores, protein, phosphorus, potassium, magnesium, and calcium intake was energy-adjusted using the residual method [22]. For both PRAL and NEAP scores, positive and negative scores reflect a higher acid- and alkaline-forming potential of diets, respectively.

### Assessment of insulin resistance

Blood samples from each participant were collected at the baseline examination after an 8-h overnight fast. Fasting glucose and fasting insulin levels were measured by a hexokinase method and radioimmunoassay, respectively. The insulin resistance index was calculated based on the homeostasis model assessment of insulin resistance (HOMA-IR) with the following formula: [fasting insulin (μIU/mL) × fasting glucose (mmol/mL)] / 22.5 [23]. Insulin resistance was defined as HOMA-IR > 2.0 [24, 25], and this value was close to the 75th percentile of our analytical sample. The endpoint for this analysis was the first occurrence of insulin resistance, and those did not occur insulin resistance were censored at date of the last follow-up examination.

### Statistical analyses

All statistical analyses were performed with SAS software version 9.4 (SAS Institute, Inc., Cary, NC, USA). A two-sided *P*-value of < 0.05 was considered statistically significant. Baseline characteristics and dietary intakes according to quartiles of PRAL and NEAP scores were described as n (%) for categorical variables and means ± standard deviations (SD) for continuous variables. Dietary acid load scores (PRAL and NEAP) were analyzed as quartiles and continuous variables. Three multivariable Cox proportional hazard models were used to estimate the hazard ratios (HRs) and 95% confidence intervals (CIs) of insulin resistance risk according to diet-induced acid load: model 1 was adjusted for sex (men or women) and age (years); model 2 was additionally adjusted for area of residence (Ansan or Ansung), education level (≤elementary school, middle/high school, or ≥ college), smoking status (never, past, or current), alcohol consumption (g/day), body mass index (BMI, kg/m^2^), fasting blood glucose (mg/dL), estimated glomerular filtration rate (eGFR, mL/min/1.73 m^2^), physical activity (metabolic equivalent task [MET]-hour/week; weekly MET hours were calculated by multiplying the reported activity hours performed by the MET value of the activity), family history of diabetes (yes or no), and history of hypertension or hyperlipidemia (yes or no); and model 3 was further adjusted for daily total energy intake and energy-adjusted carbohydrate, fat, and dietary fiber intake. Tests for trend linearity were conducted with the Wald test by considering the median values of each quartile as continuous variables in the analytic models. To explore the possible effect modifiers, we further performed analyses stratified by sex (men vs. women), age (< 50 years vs. ≥50 years), presence of obesity (BMI < 25 kg/m^2^ vs. BMI ≥25 kg/m^2^), and physical activity levels (low vs. high) in separate models.

## Results

### Characteristics of the study population

Among the study population, 49.9% were women, and the mean age was 51.5 years. The median PRAL and NEAP scores were 5.9 mEq/day (range: − 92.5–42.5 mEq/day) and 43.5 mEq/day (range: 6.6–104.5 mEq/day), respectively, and these two scores were highly correlated (*r* = 0.93, *P*-value < 0.01). Baseline characteristics of the study participants according to PRAL score are shown in Table 1. Compared with those in the lowest quartile of PRAL scores, individuals with higher scores were more likely to be men, younger, live in an urban area, and have a higher education (all, *P* < 0.01). Moreover, high proportions of participants who consume diets with high acid-forming potential were current smokers and exercised less.

**Table 1:** Characteristics of study participants at baseline by quartile of potential renal acid load, the Korean Genome and Epidemiology Study (Ansan-Ansung)

	Total	Potential renal acid load (PRAL)				*P*_trend_
		Q1 (lowest)	Q2	Q3	Q4 (highest)	
*n*	5406	1351	1352	1352	1351	
Median PRAL, mEq/d	5.9	−7.2	3.0	8.5	14.7	
NEAP, mEq/d	43.7 ± 10.5^a^	31.0 ± 5.3	40.6 ± 3.1	46.7 ± 3.4	56.5 ± 6.9	< 0.0001
Urine pH	5.7 ± 0.9	5.7 ± 0.9	5.7 ± 0.9	5.7 ± 0.8	5.6 ± 0.8	0.0011
Sex						< 0.0001
Men	2707 (50.07)	546 (40.41)	664 (49.11)	679 (50.22)	818 (60.55)	
Women	2699 (49.93)	805 (59.59)	688 (50.89)	673 (49.78)	533 (39.45)	
Age, yrs	51.5 ± 8.7	54.0 ± 8.9	52.0 ± 8.9	50.7 ± 8.4	49.4 ± 7.9	< 0.0001
Area of residence						< 0.0001
Ansung (rural)	2545 (47.08)	929 (68.76)	653 (48.30)	503 (37.20)	460 (34.05)	
Ansan (urban)	2861 (52.92)	422 (31.24)	699 (51.70)	849 (62.80)	891 (65.95)	
Education level						< 0.0001
≤ elementary school	1684 (31.15)	624 (46.19)	440 (32.54)	361 (26.70)	259 (19.17)	
middle/high school	2975 (55.03)	617 (45.67)	751 (55.55)	785 (58.06)	822 (60.84)	
≥ college	747 (13.82)	110 (8.14)	161 (11.91)	206 (15.24)	270 (19.99)	
Smoking status						< 0.0001
Never	3109 (57.51)	888 (65.73)	781 (57.77)	786 (58.14)	654 (48.41)	
Past	849 (15.70)	154 (11.40)	222 (16.42)	222 (16.42)	251 (18.58)	
Current	1448 (26.79)	309 (22.87)	349 (25.81)	344 (25.44)	446 (33.01)	
Alcohol consumption, g/d	9.6 ± 22.1	7.5 ± 20.3	8.9 ± 23.0	9.2 ± 22.0	12.7 ± 22.6	0.2260
Body mass index, kg/m^2^	24.0 ± 2.9	24.0 ± 3.0	24.0 ± 2.9	24.0 ± 2.9	23.9 ± 2.9	0.2476
Total physical activity, MET-hr/wk	169.2 ± 104.5	189.8 ± 113.6	175.2 ± 108.2	153.9 ± 96.3	157.8 ± 95.0	< 0.0001
Family history of diabetes						0.0053
Yes	562 (10.40)	115 (8.51)	131 (9.69)	166 (12.28)	150 (11.10)	
No	4844 (89.60)	1236 (91.49)	1221 (90.31)	1186 (87.72)	1201 (88.90)	
History of hypertension						< 0.0001
Yes	1410 (26.08)	405 (29.98)	367 (27.14)	330 (24.41)	308 (22.80)	
No	3996 (73.92)	946 (70.02)	985 (72.86)	1022 (75.59)	1043 (77.20)	
History of hyperlipidemia						0.0670
Yes	1251 (23.14)	300 (22.21)	300 (22.19)	313 (23.15)	338 (25.02)	
No	4155 (76.86)	1051 (77.79)	1052 (77.81)	1039 (76.85)	1013 (74.98)	

*Q* quartile, *PRAL* potential renal acid load, *NEAP* net endogenous acid production, *MET* metabolic equivalent task

^a^Values indicate the number (percentage) for categorical variables and mean ± standard deviation for continuous variables

High dietary acid load scores were significantly associated with lower intakes of total energy, carbohydrate, plant protein, dietary fiber, potassium, calcium, and magnesium and higher intakes of total fat, total protein, animal protein, and phosphorus (all, *P* < 0.01) (Table 2). Diets contributing to high acid load were characterized by higher consumption of grains/grain products, meat, fish/shellfish, and soft drinks and lower consumption of vegetables, fruits, and dairy products (all, *P* < 0.05). The baseline characteristics and nutrient and food intakes of study participants showed similar trends among NEAP score quartiles as shown in Supplementary Table 1 and 2 (Additional file 1).

**Table 2:** Nutrient and food group intake by quartile of potential renal acid load, the Korean Genome and Epidemiology Study (Ansan-Ansung)

	Potential renal acid load (PRAL)				*P*_trend_
	Q1 (lowest)	Q2	Q3	Q4 (highest)	
Nutrient intake					
Energy, kcal/d	2030 ± 686^a^	2027 ± 631	1949 ± 576	2020 ± 599	0.0002
% Energy from carbohydrate	76.1 ± 5.6	73.7 ± 5.5	72.8 ± 5.8	68.1 ± 7.0	< 0.0001
% Energy from fat	11.2 ± 4.5	12.5 ± 4.6	12.9 ± 4.8	15.9 ± 5.5	< 0.0001
% Energy from total protein	12.0 ± 2.1	12.2 ± 2.1	12.4 ± 2.2	14.0 ± 2.6	< 0.0001
% Energy from plant protein	8.3 ± 1.3	8.1 ± 1.2	7.9 ± 1.1	7.5 ± 1.2	< 0.0001
% Energy from animal protein	3.7 ± 2.2	4.2 ± 2.3	4.5 ± 2.3	6.5 ± 3.0	< 0.0001
Dietary fiber, g/d	19.2 ± 6.9	13.8 ± 4.4	11.9 ± 4.4	10.8 ± 4.0	< 0.0001
Phosphorous, mg/d	959.5 ± 178.9	942.4 ± 167.0	935.6 ± ±174.2	994.0 ± 186.5	0.0007
Potassium, mg/d	2995 ± 567	2446 ± 402	2219 ± 411	2149 ± 416	< 0.0001
Calcium, mg/d	531.9 ± 190.5	476.8 ± 158.9	447.9 ± 164.4	444.4 ± 164.7	< 0.0001
Magnesium, mg/d	178.0 ± 58.4	147.2 ± 48.8	135.8 ± 50.5	136.2 ± 48.9	< 0.0001
Food group consumption, g/d					
Grains and grain products	743.0 ± 251.6	794.3 ± 245.4	790.1 ± 243.4	771.1 ± 227.9	0.0027
Rice	663.6 ± 234.9	696.0 ± 220.7	692.3 ± 229.9	650.2 ± 201.4	0.8833
Vegetables	495.3 ± 248.3	337.5 ± 156.8	252.6 ± 127.9	224.4 ± 124.8	< 0.0001
Fruits	407.6 ± 395.2	207.4 ± 182.5	145.0 ± 133.5	118.9 ± 107.6	< 0.0001
Meat	40.6 ± 39.9	49.3 ± 47.8	50.9 ± 45.8	85.5 ± 73.5	< 0.0001
Fish and shellfish	32.4 ± 32.6	35.9 ± 37.9	37.6 ± 34.5	57.7 ± 51.0	< 0.0001
Milk and dairy products	111.0 ± 139.6	114.0 ± 133.5	107.9 ± 118.1	99.8 ± 115.8	0.0079
Soft drinks	19.5 ± 49.3	23.3 ± 52.2	22.9 ± 48.1	29.7 ± 51.4	0.0215

*Q* quartile, *PRAL* potential renal acid load

^a^Mean ± standard deviation (all such values)

### Dietary acid load and insulin resistance

During a mean follow-up of 7.4 years (39,991 person-years), 3449 new cases of insulin resistance (defined as HOMA-IR > 2.0) were identified. The results showed that highest quartile relative to the lowest quartile of PRAL and NEAP scores were associated with 14% (HR: 1.14, 95% CI: 1.03–1.25, *P*_trend_ = 0.0155) and 14% (HR: 1.14, 95% CI: 1.03–1.26, *P*_trend_ = 0.0247) higher risk of insulin resistance, respectively, after controlling for demographic characteristics, smoking and drinking status, fasting blood glucose levels, eGFR, and co-morbidities (Table 3). Associations between dietary acid load scores and insulin resistance risk were stronger in the models with additional adjustments for dietary factors, such as total energy intake and energy-adjusted intakes of carbohydrate, fat, and dietary fiber. Compared with those in the lowest PRAL and NEAP score quartiles, those in the higher quartiles experienced 30% (HR: 1.30, 95% CI: 1.13–1.48, *P*_trend_ = 0.0002) and 30% (HR: 1.30, 95% CI: 1.14–1.49, *P*_trend_ = 0.0008) higher risks of insulin resistance, respectively. In the fully adjusted models, an increase by 1 SD in PRAL and NEAP scores was associated with 13% (HR per SD: 1.13, 95% CI: 1.06–1.20) and 10% (HR per SD: 1.10, 95% CI: 1.05–1.16) increases in insulin resistance risk, respectively.

**Table 3:** Adjusted HRs (with 95% CIs) for insulin resistance by dietary acid load scores, the Korean Genome and Epidemiology Study (Ansan-Ansung)^a^

	Dietary acid load scores				*P*_trend_^b^	Per 1 SD increase
	Q1 (lowest)	Q2	Q3	Q4 (highest)		
***PRAL***						
Median, mEq/d	−7.1	3.1	8.5	14.7		5.9
Person-years	10,232	9935	9895	9929		39,991
Incident cases (*n*)	841	885	859	864		3449
Rate per 1000 person-years	82.2	89.1	86.8	87.0		86.2
	HR (95% CI)	HR (95% CI)	HR (95% CI)	HR (95% CI)		HR (95% CI)
Model 1	1.00	1.09 (0.99–1.20)	1.07 (0.97–1.18)	1.08 (0.98–1.19)	0.1193	1.02 (0.98–1.05)
Model 2	1.00	1.13 (1.02–1.24)	1.10 (1.00–1.21)	1.14 (1.03–1.25)	0.0155	1.04 (1.00–1.07)
Model 3	1.00	1.22 (1.09–1.35)	1.21 (1.08–1.36)	1.30 (1.13–1.48)	0.0002	1.13 (1.06–1.20)
***NEAP***						
Median, mEq/d	32.0	40.5	46.5	55.4		43.5
Person-years	10,148	9943	9959	9941		39,991
Incident cases (*n*)	841	879	864	865		3449
Rate per 1000 person-years	82.9	88.4	86.8	87.0		86.2
	HR (95% CI)	HR (95% CI)	HR (95% CI)	HR (95% CI)		HR (95% CI)
Model 1	1.00	1.08 (0.98–1.19)	1.07 (0.97–1.17)	1.08 (0.98–1.19)	0.1850	1.02 (0.99–1.06)
Model 2	1.00	1.15 (1.04–1.27)	1.09 (0.99–1.21)	1.14 (1.03–1.26)	0.0247	1.04 (1.00–1.07)
Model 3	1.00	1.23 (1.11–1.37)	1.20 (1.07–1.35)	1.30 (1.14–1.49)	0.0008	1.10 (1.05–1.16)

HR, hazard ratio; CI, confidence interval; Q, quartile; SD, standard deviation; PRAL, potential renal acid load; NEAP, net endogenous acid production; MET, metabolic equivalent task

^a^Model 1 was adjusted for sex (men or women) and age (years); model 2 was additionally adjusted for area of residence (Ansan or Ansung), education level (≤elementary school, middle/high school, or ≥ college), smoking status (never, past, or current), alcohol consumption (g/day), body mass index (kg/m^2^), physical activity (MET-hour/week), fasting blood glucose level (mg/dL), estimated glomerular filtration rate (mL/min/1.73 m^2^), family history of diabetes (yes or no), and history of hypertension or hyperlipidemia (yes or no); model 3 was additionally adjusted for total energy intake (kcal/day) and energy-adjusted carbohydrate, fat, and dietary fiber intakes (g/day)

^b^Tests for trend linearity were conducted with the Wald test by considering the median values of each quartile as continuous variables in the analytic models

### Stratified analyses

We examined whether the prospective associations between dietary acid load scores and insulin resistance risk differed with other type 2 diabetes risk factors, such as sex, age, obesity (BMI ≥25 kg/m^2^), and physical activity level (Table 4). Slightly stronger associations were observed among men, adults under 50 years old, obese participants, and participants with low physical activity levels. Men in the highest PRAL score quartile had a 41% higher risk of insulin resistance (HR: 1.41, 95% CI: 1.17–1.72, *P*_trend_ = 0.0017) compared with those in the lowest quartile. Similar findings were found for NEAP scores (HR for fourth vs. first quartile: 1.31, 95% CI: 1.07–1.59, *P*_trend_ = 0.0111). PRAL and NEAP scores were positively associated with insulin resistance among participants < 50 years old (HR for fifth vs. first quartile of PRAL: 1.42, 95% CI: 1.16–1.73, *P*_trend_ = 0.0012; HR for fifth vs. first quartile of NEAP: 1.43, 95% CI: 1.17–1.75, *P*_trend_ = 0.0024), but not among those ≥50 years of age. We observed stronger risk estimates among obese individuals compared with those without obesity across all quartiles of PRAL (HR: 1.36, 95% CI: 1.11–1.67, *P*_trend_ = 0.0103) and NEAP (HR: 1.33, 95% CI: 1.08–1.63, *P*_trend_ = 0.0150) scores. After stratifying participants by physical activity levels, stronger associations between dietary acid load scores and insulin resistance risk were found for those engaging in low-level exercise than with high-level exercise (HR for fifth vs. first quartile of PRAL: 1.33, 95% CI: 1.10–1.62, *P*_trend_ = 0.0078; HR for fifth vs. first quartile of NEAP: 1.36, 95% CI: 1.11–1.66, *P*_trend_ = 0.0106).

**Table 4:** Adjusted HRs (with 95% CIs) for insulin resistance by dietary acid load scores by sex, age, BMI, and physical activity levels at baseline, the Korean Genome and Epidemiology Study (Ansan-Ansung)^a^

	Dietary acid load scores				*P*_trend_^b^
	Q1 (lowest)	Q2	Q3	Q4 (highest)	
	HR (95% CI)	HR (95% CI)	HR (95% CI)	HR (95% CI)	
***PRAL***					
Sex					
Men (*n* = 2707)	1.00	1.25 (1.07–1.48)	1.16 (0.97–1.38)	1.41 (1.17–1.72)	0.0017
Women (*n* = 2699)	1.00	1.20 (1.04–1.39)	1.28 (1.10–1.50)	1.13 (0.94–1.37)	0.0431
Age					
< 50 yrs. (*n* = 2742)	1.00	1.24 (1.06–1.46)	1.24 (1.04–1.47)	1.42 (1.16–1.73)	0.0012
≥ 50 yrs. (*n* = 2664)	1.00	1.20 (1.04–1.39)	1.20 (1.03–1.41)	1.15 (0.96–1.39)	0.0683
Presence of obesity^c^					
Yes (*n* = 1892)	1.00	1.28 (1.09–1.51)	1.15 (0.96–1.37)	1.36 (1.11–1.67)	0.0103
No (*n* = 3514)	1.00	1.18 (1.03–1.37)	1.29 (1.10–1.50)	1.28 (1.07–1.52)	0.0043
Physical activity level^d^					
Low (*n* = 2735)	1.00	1.29 (1.10–1.51)	1.26 (1.06–1.50)	1.33 (1.10–1.62)	0.0078
High (*n* = 2671)	1.00	1.16 (1.00–1.34)	1.18 (1.00–1.38)	1.26 (1.05–1.52)	0.0126
***NEAP***					
Sex					
Men (*n* = 2707)	1.00	1.16 (0.99–1.37)	1.11 (0.93–1.32)	1.31 (1.07–1.59)	0.0111
Women (*n* = 2699)	1.00	1.20 (1.03–1.40)	1.26 (1.07–1.49)	1.22 (1.01–1.47)	0.0355
Age					
< 50 yrs. (*n* = 2742)	1.00	1.27 (1.08–1.50)	1.23 (1.03–1.48)	1.43 (1.17–1.75)	0.0024
≥ 50 yrs. (*n* = 2664)	1.00	1.21 (1.05–1.39)	1.19 (1.02–1.39)	1.16 (0.96–1.40)	0.1253
Presence of obesity^c^					
Yes (*n* = 1892)	1.00	1.18 (1.00–1.39)	1.10 (0.93–1.32)	1.33 (1.08–1.63)	0.0150
No (*n* = 3514)	1.00	1.26 (1.09–1.45)	1.30 (1.11–1.52)	1.28 (1.07–1.53)	0.0196
Physical activity level^d^					
Low (*n* = 2735)	1.00	1.27 (1.08–1.49)	1.22 (1.03–1.45)	1.36 (1.11–1.66)	0.0106
High (*n* = 2671)	1.00	1.20 (1.04–1.39)	1.19 (1.01–1.40)	1.23 (1.03–1.48)	0.0500

*HR* hazard ratio, *CI* confidence interval, *Q* quartile, *PRAL* potential renal acid load, *NEAP* net endogenous acid production, *BMI* body mass index, *MET* metabolic equivalent task

^a^All models were adjusted for sex (men or women), age (years), area of residence (Ansan or Ansung), education level (≤elementary school, middle/high school, or ≥ college), smoking status (never, past, or current), alcohol consumption (g/day), body mass index (kg/m^2^), physical activity (MET-hour/week), fasting blood glucose level (mg/dL), estimated glomerular filtration rate (mL/min/1.73 m^2^), family history of diabetes (yes or no), history of hypertension or hyperlipidemia (yes or no), total energy intake (kcal/day), and energy-adjusted carbohydrate, fat, and dietary fiber intake (g/day)

^b^Tests for trend linearity were conducted with the Wald test by considering the median values of each quartile as continuous variables in the analytic models

^c^Obesity was defined as BMI ≥25 kg/m^2^

^d^Physical activity levels were categorized in two groups (low vs. high) based on activity level quartile. Participants in the lower two quartiles of physical activity level were classified as the low-physical-activity group, and those in the upper two quartiles were classified as the high-physical-activity group

## Discussion

In our large population-based cohort study, diets with high acid-forming potential (reflected by high PRAL and NEAP scores) was positively associated with the future risk of insulin resistance over the mean follow-up duration of 7.3 years. Study participants in the highest PRAL score quartile had a 1.30-fold higher risk of insulin resistance relative to lowest-quartile individuals; similar risk estimates were observed for NEAP scores. Consistent with these observations, the risk of developing insulin resistance was significantly increased as PRAL and NEAP increased, respectively. Additionally, we found that participants with alkaline diets consumed more dietary fiber, vegetables, fruits, and dairy products than did participants with acidic diets. This observation is consistent with previous findings indicating that the consumption of vegetables and fruits, known to have greater alkalizing potential [26], reduced the risk of type 2 diabetes [27, 28]. Participants with higher alkalizing diets also had higher dietary fiber intake, which has been reported to aid in ameliorating glucose metabolism and delaying type 2 diabetes development [29, 30].

Our findings support those from previous epidemiological studies that reported significant associations between dietary acid load scores and increased insulin resistance risks. In a prospective Iranian cohort study, the multivariable-adjusted odds ratios (ORs) for insulin resistance risk were 2.81 (95% CI: 1.32–5.97, *P*_trend_ < 0.01) and 2.18 (95% CI: 1.03–4.61, *P*_trend_ < 0.05) for the highest quartile of PRAL and NEAP scores compared with the lowest quartile, respectively [31]. Similarly, cross-sectional studies of Japanese [32] and Danish [15] populations found a positive association between dietary acid load scores and HOMA-IR scores. In contrast to our results, a prospective study of 911 Swedish men found no associations between PRAL and NEAP scores with insulin sensitivity or β-cell function [33]. These discrepancies may be partially due to the different characteristics of the study participants; while the Swedish study targeted only 70 to 71-year-old men, our study included both men and women between 40 and 69 years of age.

The biological mechanisms of the observed associations between diet-induced metabolic acidosis and insulin resistance remain unclear, but there are several potential explanations. First, low blood pH could alter insulin binding affinity with its receptors [34]. Disrupting insulin binding to receptors inhibits the initial step of the insulin signaling pathway, which may result in reduced glucose uptake by muscle tissues and may exacerbate β-cell function, leading to insulin resistance and type 2 diabetes [35]. Second, the mechanism by which metabolic acidosis increases insulin resistance risk involves cortisol, a hormone involved in counter-regulating insulin [36]. As the hydrogen ion concentration increases, the adrenal cortex is stimulated to secrete cortisol; chronically elevated cortisol levels may induce insulin resistance [37]. Third, metabolic acidosis suppresses adiponectin gene expression and lowers circulating levels of adiponectin, which functions as an insulin sensitizer [38], and low adiponectin levels were associated with increases in HOMA-IR score and insulin resistance risk [39–41].

When we investigated the effects of sex, age, obesity, and physical activity level on the associations between dietary acid load scores and insulin resistance risk, stronger associations were observed among men, comparatively younger adults (< 50 years of age), and individuals with obesity or low physical activity levels. In agreement with previous findings [15, 42], we found that the association between dietary acid load scores and insulin resistance risk disappeared or was attenuated in women, whereas this association was strengthened in men as compared with the estimates of main effect models. Sex-specific findings may be due to sex-related differences in kidney function. Better kidney function, indicating a greater capacity for maintenance of acid-base balance, in women than in men [43, 44] may lead to weak associations between dietary acid load scores and future insulin resistance risk in women.

Based on age-stratified analysis, both PRAL and NEAP scores were significantly associated with the risk of insulin resistance development among adults < 50 years old, but not among adults ≥50 years of age. These results are partially supported by two previous prospective studies conducted in the US [14] and Japan [42], in which younger participants consumed higher acid-forming potential diets and had stronger associations between PRAL and NEAP scores and type 2 diabetes risk compared with older participants. No associations between dietary acid load scores and insulin resistance risk were found among older adults; the reason for this remains unclear. Akter et al. [42] suggested that the lack of associations in older adults may be due to a masking effect by other stronger risk factors, such as co-morbidities. This explanation could be applied to our study population, which had significantly higher prevalence of hypertension (< 50 years: 15.4% vs. ≥50 years: 29.4%, *P*-value < 0.0001) or hyperlipidemia (< 50 years: 22.1% vs. ≥50 years: 25.8%, *P*-value = 0.0084) in older adults than in younger adults.

Although no associations between dietary acid load scores and insulin resistance among individuals without obesity were observed in our study, there were 36% and 33% increases in risk estimates for obese individuals in the highest quartile of PRAL and NEAP scores, respectively. Similarly, study participants with low physical activity in the highest PRAL and NEAP score quartiles showed 33% and 36% higher risks of insulin resistance, respectively than those of the lowest quartiles. In accordance with clinical guidelines [45], our results suggest that the beneficial effects of healthy weight maintenance and increased physical activity may mitigate the detrimental effect of diet-induced metabolic acidosis on the future risk of insulin resistance.

This study had some potential limitations. First, the study population included middle-aged and elderly Koreans living in specific regions of Korea, which may limit our ability to generalize our findings to the entire Korean population encompassing a large age range. Second, we cannot rule out the possibilities of uncontrolled or residual confounding due to the nature of observational studies, although we adjusted for a wide variety of potential covariates. Third, we were unable to validate dietary acid load scores against acid-base balance markers of the human body, such as bicarbonate and the serum anion gap, due to unavailability of data. However, it was previously demonstrated that both PRAL and NEAP scores were highly correlated with metabolic acid load measured from 24-h urine samples [7, 8]. Additionally, we found that the pH values of the spot urine samples were significantly lower among participants in the highest PRAL score quartile than those in the lowest quartile, thus supporting that dietary acid load scores are applicable for estimating acid loads from overall diets of Koreans.

The present study also has several strengths. This was the first study to examine the prospective relationship between diet-induced acidosis and insulin resistance in the Korean population based on a large-scale prospective cohort study with a long follow-up period. We used dietary acid load scores derived from usual dietary intake assessed by validated FFQs, which better reflected long-term diet-induced metabolic acidosis. Furthermore, previous studies did not fully consider dietary factors as confounders, whereas we used models that were adjusted for various type 2 diabetes-related dietary components. Consequently, this study found clear associations between dietary acid load scores and insulin resistance that were independent of various other dietary factors. Particularly, we found significant associations between dietary acid load scores and future insulin resistance risks in Korean adults that have a relatively low protein intake. Moreover, we focused on sources of protein intake and their relationships with dietary acid load scores, which have not been reported by previous studies. Among countries, there were substantial differences in protein intake from animal and plant sources. Our study participants acquired more energy from plant protein (8.0%) and less from total (12.7%) and animal (4.7%) protein as compared to American populations (18.1–18.9%, 13.0–15.1%, and 5.0–7.3% energy from total, animal, and plant protein, respectively) [46]. The results of the current study showed that PRAL scores were inversely associated with plant protein intake but positively associated with total and animal protein intake in middle-aged and older Korean adults. Our results are consistent with findings from a recent meta-analysis of 11 cohort studies that demonstrated that type 2 diabetes risk was negatively associated with plant protein and positively associated with total and animal protein [47]. Future investigations are needed to explore the effects of different protein intake sources on changes in diet-induced acidosis and associations with the risk of metabolic abnormalities.

## Conclusions

In summary, the results of this large prospective study support the conclusion that diet-induced metabolic acidosis is associated with an increased risk of insulin resistance, irrespective of other type 2 diabetes risk factors. Moreover, we observed effect modifiers of associations between dietary acid load scores and insulin resistance, suggesting stronger effects among men, those < 50 years of age, obese individuals, or individuals with low physical activity levels. Our findings suggested that consuming diets with acid-base imbalance may contribute to develop future insulin resistance, even in Korean populations with a relatively low protein intake. Randomized trials are needed to confirm the effects of the overall diet and its components on acid-base equilibrium and insulin resistance.

## Supplementary Information

**Additional file 1: Supplementary Table 1.** Characteristics of study participants at baseline by quartile of net endogenous acid production, the Korean Genome and Epidemiology Study (Ansan-Ansung). **Supplementary Table 2.** Nutrient and food group intake by quartile of net endogenous acid production, the Korean Genome and Epidemiology Study (Ansan-Ansung).

## Data Availability

Data sharing is not applicable to this article as no novel datasets were generated or analyzed during the current study. Please contact the author for data requests.

## Abbreviations

BMI: Body mass index
CI: Confidence interval
eGFR: Estimated glomerular filtration rate
FFQ: Food frequency questionnaire
HOMA-IR: Homeostasis model assessment of insulin resistance
HR: Hazard ratios
KoGES: Korean Genome and Epidemiology Study
MET: Metabolic equivalent task
NEAP: Net endogenous acid production
OR: Odds ratio
PRAL: Potential renal acid load
SD: Standard deviation
