# Supplementary material for: Positive association between dietary acid load and future insulin resistance risk: findings from the Korean Genome and Epidemiology Study
Source: Nutr J. 2020 Dec 8;19:137. doi: 10.1186/s12937-020-00653-6 (PMC7724722; doi:10.1186/s12937-020-00653-6)
Supplement: Supplementary file 1 — Additional file 1: Supplementary Table 1. Characteristics of study participants at baseline by quartile of net endogenous acid production, the Korean Genome and Epidemiology Study (Ansan-Ansung). Supplementary Table 2. Nutrient and food group intake by quartile of net endogenous acid production, the Korean Genome and Epidemiology Study (Ansan-Ansung). [file 12937_2020_653_MOESM1_ESM.docx]

Supplementary Table 1. Characteristics of study participants at baseline by quartile of net endogenous acid production, the Korean Genome and Epidemiology Study (Ansan-Ansung)

|  | Total |  | Net endogenous acid production (NEAP) | | | | | | |  | *P*_trend_ |
| --- | --- | --- | --- | --- | --- | --- | --- | --- | --- | --- | --- |
|  |  |  | Q1 (lowest) |  | Q2 |  | Q3 |  | Q4 (highest) |  |  |
| *n* | 5,406 |  | 1,351 |  | 1,352 |  | 1,352 |  | 1,351 |  |  |
| Median NEAP, mEq/d | 43.5 |  | 32.0 |  | 40.5 |  | 46.5 |  | 55.4 |  |  |
| PRAL, mEq/d | 4.9 ± 12.2^a^ |  | 4.9 ± 12.2 |  | -9.7 ± 10.4 |  | 3.4 ± 3.5 |  | 9.2 ± 4.6 |  | <0.0001 |
| Urine pH | 5.7 ± 0.91 |  | 5.7 ± 0.9 |  | 5.7 ± 0.9 |  | 5.7 ± 0.9 |  | 5.7 ± 0.9 |  | 0.0008 |
| Sex |  |  |  |  |  |  |  |  |  |  | <0.0001 |
| Men | 2,707 (50.07) |  | 2707 (50.07) |  | 521 (38.56) |  | 640 (47.34) |  | 689 (50.96) |  |  |
| Women | 2,699 (49.93) |  | 2699 (49.93) |  | 830 (61.44) |  | 712 (52.66) |  | 663 (49.04) |  |  |
| Age, yrs | 51.5 ± 8.7 |  | 51.5 ± 8.7 |  | 54.4 ± 8.9 |  | 51.7 ± 8.8 |  | 50.5 ± 8.2 |  | <0.0001 |
| Area of residence |  |  |  |  |  |  |  |  |  |  | <0.0001 |
| Ansung (rural) | 2,545 (47.08) |  | 2545 (47.08) |  | 911 (67.43) |  | 621 (45.93) |  | 484 (35.80) |  |  |
| Ansan (urban) | 2,861 (52.92) |  | 2861 (52.92) |  | 440 (32.57) |  | 731 (54.07) |  | 868 (64.20) |  |  |
| Education level |  |  |  |  |  |  |  |  |  |  | <0.0001 |
| ≤elementary school | 1,684 (31.15) |  | 1684 (31.15) |  | 626 (46.34) |  | 449 (33.21) |  | 335 (24.78) |  |  |
| middle/high school | 2,975 (55.03) |  | 2975 (55.03) |  | 623 (46.11) |  | 732 (54.14) |  | 805 (59.54) |  |  |
| ≥college | 747 (13.82) |  | 747 (13.82) |  | 102 (7.55) |  | 171 (12.65) |  | 212 (15.68) |  |  |
| Smoking status |  |  |  |  |  |  |  |  |  |  | <0.0001 |
| Never | 3,109 (57.51) |  | 3109 (57.51) |  | 916 (67.80) |  | 789 (58.36) |  | 786 (58.14) |  |  |
| Past | 849 (15.70) |  | 849 (15.70) |  | 145 (10.73) |  | 208 (15.38) |  | 228 (16.86) |  |  |
| Current | 1,448 (26.79) |  | 1448 (26.79) |  | 290 (21.47) |  | 355 (26.26) |  | 338 (25.00) |  |  |
| Alcohol consumption, g/d | 9.6 ± 22.1 |  | 9.6 ± 22.1 |  | 7.0 ± 20.9 |  | 8.4 ± 21.2 |  | 9.2 ± 21.6 |  | 0.0258 |
| Body mass index, kg/m^2^ | 24.0 ± 2.9 |  | 24.0 ± 2.9 |  | 24.1 ± 3.0 |  | 23.9 ± 2.9 |  | 24.1 ± 2.9 |  | 0.0717 |
| Total physical activity, MET-hr/wk | 169.2 ± 104.5 |  | 169.2 ± 104.5 |  | 189.1 ± 113.2 |  | 173.0 ± 107.1 |  | 153.3 ± 95.2 |  | <0.0001 |
| Family history of diabetes |  |  |  |  |  |  |  |  |  |  | 0.0032 |
| Yes | 562 (10.40) |  | 562 (10.40) |  | 116 (8.59) |  | 134 (9.91) |  | 153 (11.32) |  |  |
| No | 4,844 (89.60) |  | 4844 (89.60) |  | 1235 (91.41) |  | 1218 (90.09) |  | 1199 (88.68) |  |  |
| History of hypertension |  |  |  |  |  |  |  |  |  |  | <0.0001 |
| Yes | 1,410 (26.08) |  | 1410 (26.08) |  | 423 (31.31) |  | 347 (25.67) |  | 325 (24.04) |  |  |
| No | 3,996 (73.92) |  | 3996 (73.92) |  | 928 (68.69) |  | 1005 (74.33) |  | 1027 (75.96) |  |  |
| History of hyperlipidemia |  |  |  |  |  |  |  |  |  |  | 0.0273 |
| Yes | 1,251 (23.14) |  | 1251 (23.14) |  | 306 (22.65) |  | 287 (21.23) |  | 308 (22.78) |  |  |
| No | 4,155 (76.86) |  | 4155 (76.86) |  | 1045 (77.35) |  | 1065 (78.77) |  | 1044 (77.22) |  |  |
| Q, quartile; PRAL, potential renal acid load; NEAP, net endogenous acid production; MET, metabolic equivalent task.  ^a^Values indicate the number (percentage) for categorical variables and mean ± standard deviation for continuous variables | | | | | | | | | | | |

Supplementary Table 2. Nutrient and food group intake by quartile of net endogenous acid production, the Korean Genome and Epidemiology Study (Ansan-Ansung)

|  | Net endogenous acid production (NEAP) | | | | | | |  | *P*_trend_ |
| --- | --- | --- | --- | --- | --- | --- | --- | --- | --- |
|  | Q1 (lowest) |  | Q2 |  | Q3 |  | Q4 (highest) |  |  |
| Nutrient intake |  |  |  |  |  |  |  |  |  |
| Energy, kcal/d | 2035 ± 695^a^ |  | 1999 ± 592 |  | 1975 ± 608 |  | 2006 ± 597 |  | <0.0001 |
| % Energy from carbohydrate | 76.6 ± 5.3 |  | 73.5 ± 5.4 |  | 72.0 ± 6.0 |  | 68.8 ± 7.4 |  | <0.0001 |
| % Energy from fat | 10.8 ± 4.3 |  | 12.5 ± 4.5 |  | 13.5 ± 4.9 |  | 15.5 ± 5.7 |  | <0.0001 |
| % Energy from total protein | 11.8 ± 2.0 |  | 12.4 ± 2.1 |  | 12.7 ± 2.3 |  | 13.6 ± 2.8 |  | <0.0001 |
| % Energy from plant protein | 8.3 ± 1.3 |  | 8.2 ± 1.2 |  | 7.9 ± 1.1 |  | 7.5 ± 1.1 |  | <0.0001 |
| % Energy from animal protein | 3.5 ± 2.0 |  | 4.3 ± 2.3 |  | 4.9 ± 2.4 |  | 6.2 ± 3.1 |  | <0.0001 |
| Dietary fiber, g/d | 19.0 ± 6.9 |  | 14.4 ± 4.5 |  | 12.2 ± 4.3 |  | 10.4 ± 4.1 |  | <0.0001 |
| Phosphorous, mg/d | 959.3 ± 173.3 |  | 967.9 ± 171.5 |  | 956.3 ± 175.8 |  | 948.1 ± 191.9 |  | 0.0272 |
| Potassium, mg/d | 2973 ± 569 |  | 2504 ± 417 |  | 2280 ± 398 |  | 2066 ± 412 |  | <0.0001 |
| Calcium, mg/d | 519.8 ± 187.1 |  | 487.4 ± 169.9 |  | 463.7 ± 163.3 |  | 426.8 ± 157.0 |  | <0.0001 |
| Magnesium, mg/d | 176.6 ± 58.3 |  | 154.6 ± 49.2 |  | 139.7 ± 49.4 |  | 127.1 ± 49.4 |  | <0.0001 |
| Food group consumption, g/d |  |  |  |  |  |  |  |  |  |
| Grains and grain products | 750.8 ± 258.1 |  | 776.7 ± 236.6 |  | 781.1 ± 244.3 |  | 784.7 ± 230.1 |  | 0.0065 |
| Rice | 677.9 ± 243.5 |  | 687.2 ± 218.7 |  | 679.6 ± 222.8 |  | 654.6 ± 204.2 |  | 0.0160 |
| Vegetables | 475.2 ± 247.8 |  | 339.6 ± 168.5 |  | 273.2 ± 139.9 |  | 219.1 ± 121.5 |  | <0.0001 |
| Fruits | 410.2 ± 410.9 |  | 209.0 ± 183.5 |  | 153.7 ± 142.7 |  | 115.6 ± 107.0 |  | <0.0001 |
| Meat | 37.3 ± 38.0 |  | 47.7 ± 42.8 |  | 56.6 ± 50.5 |  | 83.7 ± 74.2 |  | <0.0001 |
| Fish and shellfish | 30.2 ± 29.3 |  | 37.5 ± 39.0 |  | 42.1 ± 38.3 |  | 53.3 ± 50.8 |  | <0.0001 |
| Milk and dairy products | 111.5 ± 136.2 |  | 115.5 ± 137.4 |  | 111.9 ± 121.3 |  | 91.1 ± 106.8 |  | <0.0001 |
| Soft drinks | 18.9 ± 48.3 |  | 21.8 ± 50.2 |  | 25.1 ± 51.6 |  | 28.8 ± 49.6 |  | <0.0001 |
| Q, quartile; NEAP, net endogenous acid production.  ^a^Mean ± standard deviation (all such values) | | | | | | | | | |
